# Diagnostic PCR assays to unravel food web interactions in cereal crops with focus on biological control of aphids

**DOI:** 10.1007/s10340-015-0685-8

**Published:** 2015-08-11

**Authors:** Karin Staudacher, Mattias Jonsson, Michael Traugott

**Affiliations:** Mountain Agriculture Research Unit, Institute of Ecology, University of Innsbruck, Technikerstraße 25, 6020 Innsbruck, Austria; Department of Ecology, Swedish University of Agricultural Sciences, PO Box 7044, 750 07 Uppsala, Sweden

**Keywords:** Molecular gut content analysis, Group-specific primer, Multiplex PCR, Generalist predators, Carabid beetles

## Abstract

Successful biological control of agricultural pests is dependent on a thorough understanding of the underlying trophic interactions between predators and prey. Studying trophic interactions can be challenging, particularly when generalist predators that frequently use multiple prey and interact with both pest and alternative prey are considered. In this context, diagnostic PCR proved to be a suitable approach, however at present, prey-specific PCR primers necessary for assessing such interactions across trophic levels are missing. Here we present a new set of 45 primers designed to target a wide range of invertebrate taxa common to temperate cereal crops: cereal aphids, their natural enemies such as carabid beetles, ladybeetles, lacewings, and spiders, and potential alternative prey groups (earthworms, springtails, and dipterans). These primers were combined in three ‘ready to use’ multiplex PCR assays for quick and cost-effective analyses of large numbers of predator samples. The assays were tested on 560 carabids collected in barley fields in Sweden. Results from this screening suggest that aphids constitute a major food source for carabids in cereal crops (overall DNA detection rate: 51 %), whereas alternative extraguild and intraguild prey appear to be less frequently preyed upon when aphids are present (11 % for springtails and 12 % for earthworms; 1 % for spiders and 4 % for carabids). In summary, the newly developed molecular assays proved reliable and effective in assessing previously cryptic predator–prey trophic interactions, specifically with focus on biological control of aphids. The diagnostic PCR assays will be applicable manifold as the targeted invertebrates are common to many agricultural systems of the temperate region.

**Electronic supplementary material:**

The online version of this article (doi:10.1007/s10340-015-0685-8) contains supplementary material, which is available to authorized users.

## Key message

- Biological control of pests requires a thorough understanding of food web interactions which can be unravelled via diagnostic PCR. However, there is a lack of prey-specific primers to assess predator–prey interactions across trophic levels.
- Twenty-four primer pairs for cereal aphids, non-pest alternative prey, and generalist predators are presented. Three ‘ready to use’ multiplex PCR assays employing these primers were developed and successfully applied to screen field-collected carabid beetles for prey DNA.
- The diagnostic PCR assays are applicable manifold and allow effectively assessing predator–prey trophic interactions in cereal crops and other agricultural systems.

## Introduction

Biological control of agricultural pests by natural enemies is an ecosystem service of immense economic value (Landis et al. 2008; Losey and Vaughan 2006; Östman et al. 2003). To increase the potential for biological control in modern agricultural landscapes, a thorough understanding of the interactions at play between predators and prey is urgently needed. In this context, food web approaches, relying on the understanding of trophic interaction networks, have been highlighted as providers of a functional insight into arable invertebrate communities (Bohan and Woodward 2013; Miranda et al. 2013). Arable food webs are usually highly complex and dynamic due to spatio-temporal fluctuations in predator and prey densities and coining extrinsic factors such as farming practice and habitat heterogeneity (Macfadyen et al. 2011; Rusch et al. 2010, 2014). This is especially true for food webs involving generalist predators, many of which are effective natural enemies of pests, but not always constitutive such that they regularly choose from a variety of alternative extraguild and intraguild prey (Davey et al. 2013; Eitzinger and Traugott 2011; Kuusk and Ekbom 2010; Lang 2003).

Aphids, among other insect pests, can inflict considerable damage in cereals, one of the most important crops worldwide (FAOSTAT 2012), by either directly feeding on cereal plants or by transmitting pathogens (van Emden and Harrington 2007). Cereal aphid suppression by generalist predators such as ground beetles, rove beetles, or spiders can be substantial, especially during times when aphids colonise the crop (Chiverton 1987; Ekbom et al. 1992; Östman et al. 2003; Symondson et al. 2002). The efficacy of generalist predators as aphid biocontrol agents, however, has been found to be variable and thus hard to predict. It is potentially affected by trophic interactions among predators, aphids, and non-pest prey, with either antagonistic or additive/synergistic effects on aphid control (Losey and Denno 1998; Rosenheim 2007; Roubinet et al. 2015; Straub and Snyder 2006). Consequently, a thorough understanding of such interactions is important, but largely rests with the ability to directly track the feeding links through these food webs (Bohan and Woodward 2013).

Molecular gut content analysis (MGCA) is an effective approach of studying trophic interactions, which can be applied to any prey type and is applicable to semi-digested and/or visually undiscernible prey remains (Symondson and Harwood 2014). Diagnostic PCR, a straightforward type of MGCA, allows the identification of prey at different taxonomic levels, e.g. order, family, genus, or species level, depending on the specificity of the respective PCR primers (Traugott et al. 2013). The combination of several prey-specific primer pairs in multiplex PCR assays enables targeting multiple prey taxa within a single reaction, which considerably reduces time and costs associated with analysing multiple trophic links (Sint et al. 2012). Besides, both diagnostic PCR and subsequent electrophoretic visualisation of prey-specific amplicons can be performed with standard molecular equipment and are ideally suited for processing large numbers of samples. That is a major benefit of this approach compared to sequence-based prey identification such as next-generation sequencing (NGS) techniques which include extensive bioinformatics analyses (Pompanon et al. 2012).

Along with the growing number of studies employing diagnostic PCR to assess trophic interactions of invertebrates in arable crops, the availability of PCR primers (in particular for agricultural pests) has increased steadily (e.g. King et al. 2008; Symondson 2012: summary of published primers, including invertebrate predators, pest/non-pest prey). However, at present, prey-specific primers necessary for thoroughly assessing predator–prey interactions across trophic levels are missing. For example, there is a lack of primers targeting higher taxonomic levels of prey which are of great value to generate an overview of the main trophic links in the food web (Jarman et al. 2004; Koester et al. 2013; Sint et al. 2014; Zarzoso-Lacoste et al. 2013). Moreover, family- and genus-specific primers targeting abundant generalist arthropod predators such as *Bembidion* spp., *Harpalus* spp., and spiders (Lycosidae, Linyphiidae) are not yet available. Such primers would enable investigating intraguild predation, a type of trophic interaction which is important for assessing the efficacy of natural enemies in food webs (Cardinale et al. 2003) and has been investigated molecularly in different agricultural systems (e.g. Davey et al. 2013; Harwood et al. 2007; Ingels et al. 2013; Moreno-Ripoll et al. 2014; Traugott et al. 2012).

Here, we present a new versatile set of PCR primers targeting a range of invertebrate prey taxa, comprising aphids and their natural enemies, as well as alternative extraguild prey, all of which are commonly found in temperate cereal crops. We aimed to design primers (i) which allow targeting DNA of these invertebrates at different taxonomic levels, (ii) which are highly sensitive to amplify minute quantities of prey DNA, and (iii) which offer the possibility to be combined in customised multiplex PCR assays for an efficient application in large-scale field studies to assess trophic interactions at the food web level.

To validate the practical applicability of these primers, we tested them on predator samples from barley fields in Southern Sweden. Carabid beetles which are known to consume cereal aphids (Sunderland 2002) as well as alternative extraguild and intraguild prey (Thiele 1977), were collected at colonisation of the bird cherry-oat aphid *Rhopalosiphum padi* and when aphid population densities were expected to peak. Predator gut contents were screened for prey DNA using multiplex PCR assays employing the newly developed primers and, subsequently, the food spectrum of large (>10 mm) and small carabid beetles was compared for the two sampling dates.

## Materials and methods

### Compilation of sequence databases for primer design

A comprehensive set of invertebrates, all common throughout agricultural systems in Europe, was compiled to establish DNA extracts and sequences (see below) for the development of the PCR primers (Tables 1, 2). Specimens were mainly collected in 2011/12, in cereal fields in south-central Sweden (Counties of Uppsala and Scania; collected/identified by G. Malsher) or in agricultural areas close to the University of Innsbruck (Tyrol, Austria; collected/identified by M. Traugott, N. Schallhart) and the University of Göttingen (Lower Saxony, Germany; collected/identified by I. Vollhardt). Cereal aphids were obtained from Katz Biotech AG (Baruth, Germany). All invertebrates were individually placed in 2 ml reaction tubes, freeze-killed, and afterwards stored in 70–90 % ethanol.

**Table 1:** Invertebrate taxa targeted by newly developed primers. Columns show the taxonomic affiliation of the targets and names of primers at different taxonomic levels. For each taxon, several specimens were DNA extracted and sequenced; taxa which were tested in silico only are indicated by †. For specific characteristics of primers see ESM 2

Note that for the following taxa reliable amplification with the respective primers was not possible, but taxa were used to evaluate the specificity of the primers/assays: Coccinellidae *Exochomus quadripustulatus*, carabid beetle *Nebria brevicollis*, beetle families Elateridae (*Agriotes obscurus, Hemicrepidius niger*), Cantharidae, and Silphidae, spider family Theridiidae, dipteran family Chironomidae and thrips *Aeolothrips fasciatus* and *Parthenothrips* sp.

**Table 2:** Non-target invertebrate taxa used to evaluate the specificity of the newly developed primers in PCR

The DNA of several individuals per taxon (2–3 on average) was extracted using muscle tissue to prevent any co-extraction of DNA from the gut content and/or external contaminants. In very small specimens (such as flea beetles, springtails and thrips), whole-body sections (e.g. abdomen) or the entire animal was used. For the latter, mostly individuals that were starved prior freeze-killing were used. In case the DNA sequence was of low quality and/or the DNA extract tested positive for prey DNA, the specimen was excluded from the test set.

Tissue samples were lysed in 430 µl TES buffer (0.1 M TRIS, 10 mM EDTA, 2 % SDS; pH 8) and 10 µl Proteinase K (20 mg ml^−1^), homogenised with 3 mm glass beads for 1 min at 5000 rpm using a Precellys^®^ 24 Tissue Homogenizer (Bertin Technologies, Montigny-le-Bretonneux, France), and incubated overnight at 58 °C. DNA was subsequently extracted using the BioSprint 96 DNA blood Kit (Qiagen, Hilden, Germany) running on the BioSprint 96 instrument (Qiagen) in accordance to the manufacturer’s instructions, yielding 200 µl of DNA extract per sample.

Universal invertebrate primers were employed to amplify two genes: (i) parts of the nuclear 18S rRNA gene using primers from Luan et al. (2003) (18sL0001/18sL0466 and 18sR1100) and von Dohlen and Moran (1995) for cereal aphids, as well as (ii) the 5′-end of the mitochondrial cytochrome *c* oxidase subunit I (COI) gene using the primers described in Folmer et al. (1994) (LCO1490 and HCO2198) together with an intermediate primer from Simon et al. (1994) (C1-J-1859). Amplifications were performed with standard singleplex PCR chemistry (One*Taq*^*®*^ DNA polymerase, 50 °C annealing temperature, for details see ESM 2) following the thermocycling recommendations of the manufacturer and carried out in a Mastercycler Gradient (Eppendorf, Hamburg, Germany). PCR products were separated and visualised using the automated capillary electrophoresis system QIAxcel (Qiagen). Sequencing of purified PCR products with the above-described universal primers (in both forward and reverse directions) was conducted by Eurofins MWG Operon (Ebersberg, Germany). Generated 18S and COI sequences were edited and aligned manually using BioEdit Sequence Alignment Editor v7.1.9 (Hall 1999) and representative sequences for both genes were submitted to GenBank (accession numbers listed in ESM 1; KT204317–KT204433). The two resulting sequence databases were improved and extended with sequences of closely related taxa available in GenBank (ESM 1).

### Primer design and evaluation

Primer Premier 5 (PREMIER Biosoft International, Palo Alto, USA) was used to design primers targeting invertebrate DNA at different taxonomic levels. We aimed to generate primer pairs that amplify DNA fragments not longer than 400 bp and thus being well suited to amplify semi-digested DNA (Traugott et al. 2013). To allow the combination of primer pairs in multiplex PCR assays, taxon-specific amplicons of different sizes were created. Having the choice of two genes (18S and COI) increased the possibility to design primer pairs of desired specificity and distinct amplicon length. To combine newly developed primers with published ones, the latter (springtails primers, see “Results”) were additionally analysed in Primer Premier 5 and modified to comply with our criteria for in silico evaluation, such as melting temperatures between 59 and 62 °C.

In vitro evaluation of the primers’ specificity, sensitivity, and diagnostic efficacy was performed for all primer pairs in singleplex PCRs and, in most cases, also in multiplex PCR assays; likewise, the optimisation of the PCR protocols focussed on the primer performance in both applications. Assay-specific refinements mainly involved modifications of annealing temperature and DNA extract volume, as well as deployment of PCR-enhancing agents such as Q-solution (Qiagen) and tetramethylammonium chloride (TMAC, Sigma-Aldrich, St. Louis, USA).

The specificity of the primer pairs was evaluated using a comprehensive target and non-target set of DNA extracts (all taxa tested are listed in Tables 1, 2). For evaluating the sensitivity of the primer pairs, DNA templates of the targets were generated using the universal 18S and COI primers described above to amplify fragments covering the primers’ binding sites following the procedure described in Sint et al. (2012) (for PCR details see ESM 2). The sensitivity of primer pairs in both singleplex and multiplex PCR assays was tested on twofold serially diluted DNA templates ranging from 1000 to 62.5 double-stranded (ds) copies µl^−1^ of DNA template. Note that the final number of template molecules in the PCR depends on the volume of template DNA used; for example, when 1.5 µl of template DNA of a 62.5 ds copies µl^−1^ concentration is used, the actual number of ds template molecules subjected to PCR is 93.75. To balance the sensitivity for all primer pairs used in multiplex PCR assays, the concentration of individual primers was adjusted based on amplification signal strength, i.e. the relative fluorescent units (RFUs) provided by QIAxcel (Qiagen) (see Table 3, conc. of primers in multiplex PCR). For example, the concentration of a primer pair with an initially higher amplification signal strength compared to other primers used in the assay was gradually lowered until an overall balanced sensitivity was achieved. To assess the diagnostic efficacy of the primer pairs in multiplex PCR, mixes of DNA templates targeted by the respective multiplex PCR assays were tested. These mixes contained equal ratios of DNA templates of all targets. In addition, to simulate gut content samples, whole-body DNA extracts of predators (carabid beetles *Pterostichus melanarius* and *Trechus quadristriatus*; wolf spider *Pardosa agrestis*) were spiked with ‘prey DNA’ at two concentrations, namely 250 and 125 ds copies µl^−1^ DNA template, and tested for the prey in singleplex PCRs to check for potential inhibiting influence of the predator DNA.

### Customised multiplex PCR assays and their applicability

The majority of the primers were employed in multiplex PCR assays. In particular, we aimed at establishing one multiplex PCR assay comprising group-specific primer pairs to allow the examination of the predators’ food choice on a more general level and two additional multiplex PCR assays which would each enable the detection of spiders and beetles at lower taxonomic levels. The rationale here is that, in an iterative screening process, the latter two assays could be used on samples that tested positive in the group-specific assay to provide a higher taxonomic resolution of the spider and beetle prey (i.e. a two-step procedure), and to assess intraguild predation.

The applicability of these multiplex PCR assays was tested by screening 560 carabid beetles which were collected in two spring-sown barley fields in Southern Sweden (Scania; field A N55° 48.60632 E13° 35.3829, 142 m a.s.l and field B N55° 35.38687 E13° 36.31455, 43 m a.s.l) in 2012. Sampling was conducted at aphid colonisation end of May and at the end of June when aphid peak density was expected to occur. Two plots (24 × 24 m; located opposite of each other) per field were sampled for carabids. In each plot, 20 dry pitfall traps (Ø 11.5 cm, 11 cm depth; partially filled with clay balls to impede within-trap predation events; Sunderland et al. 2005) were established in a grid with 4 m distance between each trap. At each sampling date, traps were opened at night (~20:00) and emptied after approximately 12 and 24 h. All beetles caught were individually stored in 2 ml reaction tubes without any solvent, immediately cooled at 3–5 °C in the field, and frozen at −50 °C on the same day. Additionally, aphids were counted on 50–100 randomly selected tillers within each plot at each sampling date.

Carabids were identified to species level and thereafter transferred to lysis buffer (430 and 630 µl TES for small and large, i.e. >10 mm, carabid beetles, respectively, and 10 µl Proteinase K) to extract DNA of the predator and any prey DNA present in its gut. Beetles were homogenised with glass beads (Precellys^®^, Bertin Technologies) and incubated overnight at 58 °C; DNA was subsequently extracted using the BioSprint Kit (Qiagen) (for details see above). All extractions were done in a separate pre-PCR laboratory; several negative controls (lysis buffer, with and without glass beads; on average five controls per batch of 96 samples) were included in each extraction to check for DNA carry-over contamination during all steps. Negative controls were processed following the same procedure as the one for the beetle samples and then tested with the universal COI primers (for PCR details see ESM 2).

All carabid beetles were screened with the first multiplex PCR assay (*MPI*; for PCR details see “Results”) to test for DNA of cereal aphids, alternative extraguild and intraguild prey. Specimens that tested positive for spider DNA were assigned to the *MPII spiders* multiplex PCR assay to identify the specific spider prey on family/genus level. All carabid beetles were further tested in the *MPII beetles/thrips* multiplex PCR assay to detect carabid–carabid feeding interactions. To avoid corrupting the amplification success of prey DNA, which is present in much smaller amounts than that of the consumer in the whole-body DNA extracts used, the primer pair targeting the genus of the respective beetle examined (i.e. the consumer DNA) was excluded. For example, there was no primer pair for *Pterostichus* spp. when screening *Pterostichus* spp. beetles. Three positive (artificial mixes of target DNA at low concentrations) and two negative controls (PCR-grade water instead of DNA) were run within each 96-well PCR plate to check for correct amplification and DNA carry-over contamination. All PCR products were separated and visualised using the QIAxcel system (AL320 separation method, DNA Screening Kit, Qiagen) and scored with BioCalculator (Qiagen). The detection threshold was set at 0.075 RFUs and target amplicons with signal strength above this were deemed to be positive. In two cases beetle DNA extracts tested negative in the first multiplex PCR assay and in the subsequently performed re-testing with universal primers (for PCR details see ESM 2); these two samples were excluded from the data set leaving 558 specimens for analysis. Post-screening, at least five PCR products from each prey type amplified from the carabids were DNA sequenced with the respective primers in forward direction. DNA sequences were subsequently matched with sequence databases and in each of these samples the assigned identity of the prey as detected with our diagnostic PCR approach was confirmed.

For analysis of the field-derived trophic data each sampling date was treated separately and prey DNA detection rates (i.e. proportion of carabids testing positive for a prey type) were compared between large (>10 mm) and small carabid beetles. This was done using one-sample *t* tests combined with a bootstrapping procedure including 9999 permutations (Spotfire S+8.1 for Windows, TIBCO Spotfire, Somerville, USA). The tilting confidence interval was set to 95 %, such that non-overlapping intervals indicate significant differences at *P* < 0.05. For the first sampling date (aphid colonisation), the data from the two fields were pooled, as aphid abundances were considered similar in both fields (0.34 ± 0.82 and 0.41 ± 0.97 aphids per tiller in field A and B, respectively; mean ± SD). For the second sampling date (peak aphid density), however, a field-specific analysis was additionally conducted as in field A the aphid population density at peak was estimated at 27.82 ± 16.48 aphids per tiller, whereas in field B only 0.92 ± 1.49 aphids per tiller were counted (Mann–Whitney *U* test, *U* = 44.5, *P* < 0.001; implemented in IBM SPSS 21 Statistics, IBM, Armonk, USA).

## Results

### Prey-specific primers and customised multiplex PCR assays

**Fig. 1:**
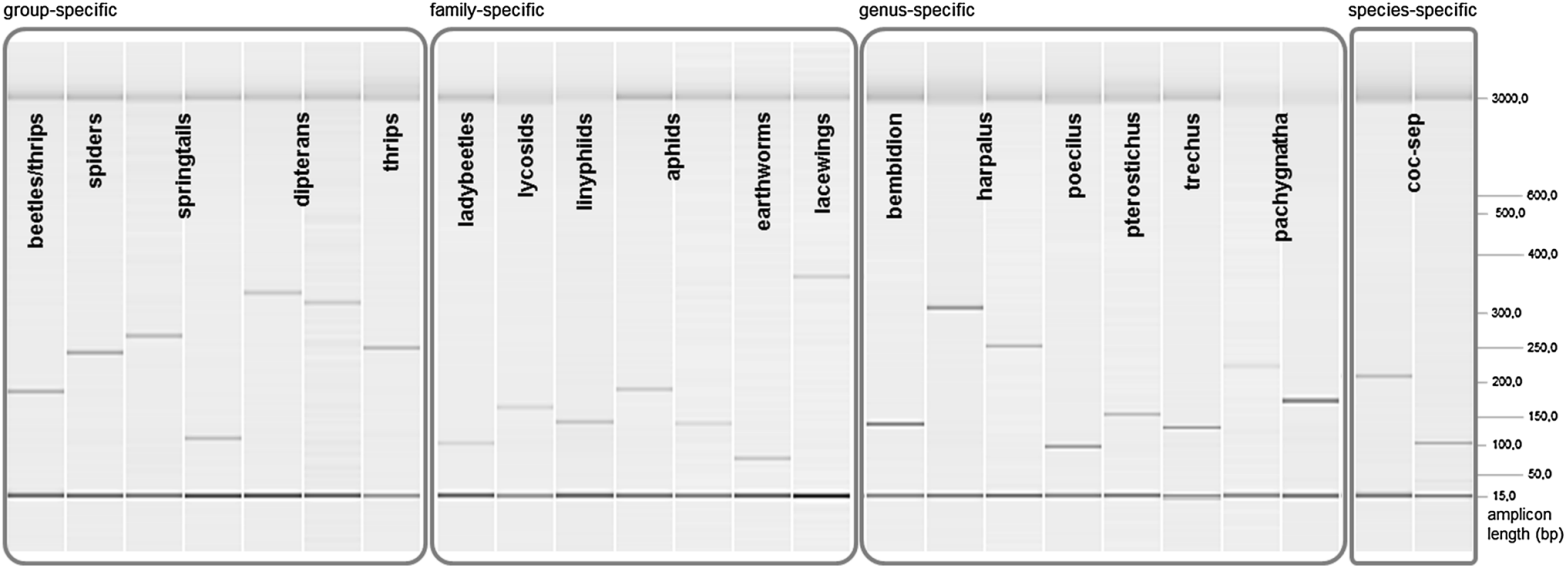
Gel image of PCR products amplified with the newly developed primers and visualised with the QIAxcel system. Different taxonomic levels of primers (group-/family-/genus- and species-specific) are indicated above boxes. For several taxa (i.e. springtails, dipterans, aphids, *Harpalus* spp., *Pachygnatha* spp., and *Coccinella septempunctata*), two versions of primer pairs amplifying different amplicon lengths are shown. An alignment marker (15 and 3000 bp) was running with each sample and a base pair scale indicates amplicon length on the right side. All targets amplified from 125/250 ds copies µl^−1^ DNA templates, except for *Pachygnatha* spp. 1000 ds copies µl^−1^ DNA template

In total, 45 PCR primers (24 primer pairs) based on either the 18S or the COI gene were designed to target a broad range of invertebrates including beetles, spiders, aphids, earthworms, springtails, dipterans, lacewings, and thrips at different levels of taxonomic resolution (Table 1). The primer pairs generate amplicons ranging between 85 and 390 bp in length and are ideally suited for amplification of semi-digested, degraded prey DNA. Moreover, two alternative primer pairs each were developed for aphids, springtails, dipterans, *Pachygnatha* spp., *Harpalus* spp., and *Coccinella septempunctata* to have both a longer and shorter amplicon providing more flexibility for combining the primer pairs in customised multiplex PCR assays (Fig. 1; Tables 1, 3). Three of the four presented primers for springtails (S411, S412, A415) have previously been published, but were slightly modified to comply with our requirements (Table 3).

Due to the high sequence similarity between beetles and thrips within the 18S primer binding regions, their DNA is amplified by the so-called beetles/thrips-primer pair. The primer pair specific for thrips (S477–A481) can be used to identify this prey group (i.e. *Frankliniella* spp. and *Limothripsdenticornis*), but there is no primer pair that only amplifies beetles. It should be noted that family-specific primers for Lycosidae and Linyphiidae are restricted to three genera each, *Pardosa*, *Trochosa*, *Alopecosa* (Lycosidae) and *Agyneta*, *Erigone*, and *Oedothorax* (Linyphiidae) (Table 1). For further details on the characteristics of the developed primers see ESM 2.

**Fig. 2:**
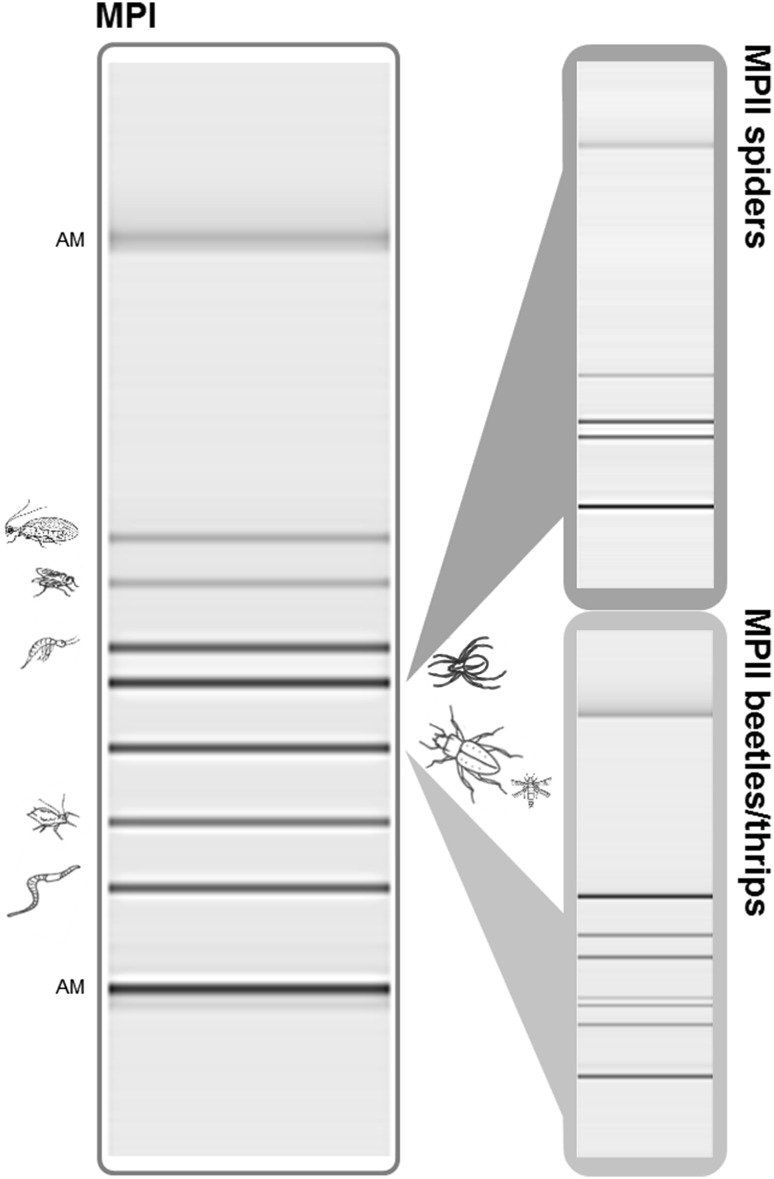
Gel image of PCR products amplified with the three customised multiplex PCR assays and visualised with the QIAxcel system. MPI (*left side*) comprises group/family-specific primers for seven taxa: beetles/thrips, spiders, aphids, earthworms, springtails, dipterans, and lacewings. MPII spiders (*upper right side*) covers two spider families, i.e. lycosids and linyphiids, as well as the genus *Pachygnatha*. MPII beetles/thrips (*lower right side*) addresses six taxa: the carabid genera *Poecilus*, *Bembidion*, *Pterostichus,* and *Harpalus*, the ladybeetle *Coccinella septempunctata* as well as thrips (*Frankliniella*, *Limothrips*). The shortest and longest fragments within each lane represent the two alignment markers (AM; 15 and 3000 bp) as indicated in the* left panel*. For amplicon lengths see Fig. 1 and Table 3. Mixes of DNA templates of targets; approximately 1000 ds copies each in PCR

The following three multiplex PCR assays were established: (i) *MPI*, a group/family-specific multiplex PCR assay covering beetles/thrips, spiders, aphids, earthworms, springtails, dipterans, and lacewings; (ii) *MPII spiders*, a family/genus-specific assay targeting lycosids, linyphiids, and *Pachygnatha* spp.; and (iii) *MPII beetles/thrips*, a group/genus/species-specific assay targeting four carabid genera, *C. septempunctata,* and thrips (Fig. 2, Table 3). The *MPI* assay was performed in a total volume of 10 µl containing 1.5 µl of DNA extract, 1× QIAGEN Multiplex PCR Master Mix (Qiagen), each primer at its corresponding concentration (Table 3), 0.5× Q-solution (Qiagen), 5 µg BSA, 30 mM TMAC (Sigma-Aldrich), and PCR-grade water to adjust the volume. Amplifications were carried out under the following thermocycling conditions: 15 min at 95 °C, 35 cycles of 30 s at 94 °C, 90 s at 63.5 °C and 90 s at 72 °C, and 10 min at 72 °C. The *MPII spiders* assay was performed in 10 µl PCRs containing 3.5 µl of DNA extract, 1× Type-it Multiplex PCR Master Mix (Qiagen), each primer at its corresponding concentration (Table 3), and 5 µg BSA. The thermocycling protocol included an initial activation step of 5 min at 95 °C, followed by 35 cycles of 30 s at 95 °C, 3 min at 61 °C and 30 s at 72 °C, and 10 min at 68 °C. The PCR protocol of the *MPII beetles/thrips* assay differed only slightly from the *MPII spiders*: 1.5 µl of DNA extract and 30 mM TMAC (Sigma-Aldrich) were used in the total volume of 10 µl (plus PCR-grade water to adjust the volume); thermocycling conditions as described above, but with an annealing temperature of 63.5 °C.

**Table 3:** Newly developed primers and the three multiplex PCR assays for assessing trophic interactions of invertebrates in cereal crops. Columns show the primer targets, primer names (S and A denote forward and reverse primers, respectively), targeted gene, primer sequences, expected amplicon lengths, detection limits, and final concentration (conc.) of each primer when used in the multiplex PCR assays (MPI, MPII spiders, and MPII beetles/thrips; if concentrations of forward and reverse primer are different, both are listed). Detection limits refer to the lowest numbers of double-stranded template molecules (copies per µl DNA template) where a detectable amplicon could be generated (i.e. signal strength ≥0.075 RFUs; QIAxcel) in singleplex and optionally multiplex PCR (in parenthesis). The primers marked with * and ** were 1:1 mixes of the two forward primer variants. S411-springtails primer was developed by the authors and published elsewhere (Roubinet et al. 2015); A415 and S412 are slightly modified versions of springtail-primers Col-gen-A246 (Sint et al. 2012) and Col3F (Kuusk and Agusti 2008), respectively (modifications apply to underlined bases). Note that for *Trechus* amplicon length varies between the two species: *T. quadristriatus*, 142 bp, *T. secalis*, 152 bp; the two closely related genera *Bembidion* and *Trechus* share the same forward primer (S468)

† Due to quality issues of the DNA template for *Pachygnatha* spp., the sensitivity of the respective primer pairs was additionally tested with highly diluted (1:1000) DNA extracts of *Pachygnatha* *clercki* where always very strong signals were produced (>2.4 RFUs)

The evaluation of primer performance with regard to specificity, sensitivity, and diagnostic efficacy in singleplex, and if applicable also multiplex PCR, was based on the above optimised protocols (for further details see ESM 2). No cross-reactions with non-target DNA were observed when primers were tested in singleplex PCRs against 156 invertebrate taxa from four classes (all taxa tested are listed in Tables 1, 2). Occasionally, we detected some longer amplicons (>800 bp) with samples of the carabid beetles *Harpalus* spp. and *Anchomenus*/*Agonum* spp., rove beetle *Philonthus* spp., earthworm *Aporrectodea* spp., and plant bug *Lygus* spp. in the *MPII spiders* assay. An approximately 220 bp sideband occasionally appeared with thrips DNA in the *MPII beetles/thrips* assay due to the combination of the forward primer for thrips (S477) and the reverse primer for *Pterostichus* spp. (A467.1). These sidebands do, however, not corrupt the diagnostic PCR as they are not interfering with the length of the target amplicons.

The primers proved to be highly sensitive in singleplex PCR: amplification of the target DNA was successful with 125 ds copies µl^−1^ DNA template and often also with as little as 62.5 ds copies µl^−1^ DNA template (i.e. signal strength ≥0.075 RFUs); only the primer pair for dipterans (version 1) and *Pachygnatha* spp. (both versions) exhibited a lower sensitivity (Table 3). The presence of predator DNA of *P. melanarius,T. quadristriatus* and *P. agrestis* did not decrease the sensitivity of the primers. In all cases, 125 ds copies µl^−1^ DNA template were sufficient to amplify the prey DNA template molecules in the spiked samples—only for the primer pairs for dipterans (version 1), thrips and *Pachygnatha* spp. (both versions) more copies were needed: 250 ds copies µl^−1^ DNA template for dipterans and thrips, and >250 ds copies µl^−1^ DNA template for *Pachygnatha* spp. All customised multiplex PCR assays (*MPI, MPII spiders, MPII beetles/thrips*) were highly sensitive as well (i.e. 125 ds copies µl^−1^ DNA template were adequate in most cases to generate amplicons well detectable in electrophoresis; Table 3). When testing mixes of DNA templates of all prey taxa targeted by a respective multiplex PCR assay, 500 ds copies on average of each target in PCR were sufficient, with the exception of *Pachygnatha* spp. in *MPII spiders,* where ~1000 ds copies of the DNA template were needed for successful amplification.

### Prey DNA detection in field-collected carabid beetles

In total, 154 large and 406 small carabids, comprising 26 species of 12 genera, were collected at the two sampling dates in the two barley fields (ESM 3) and analysed for their gut content (two individuals excluded due to failed DNA extraction). The most common large carabids were *Poecilus cupreus* (4.6 %), *Poecilus versicolor* (5.4 %), and *Pterostichus melanarius* (10.4 %); the catches of the ‘small carabids’ were dominated by *Bembidion lampros* (30.5 %) and *Bembidion tetracolum* (23 %) (percentages in parentheses refer to proportion of the total number of individuals caught).

**Fig. 3:**
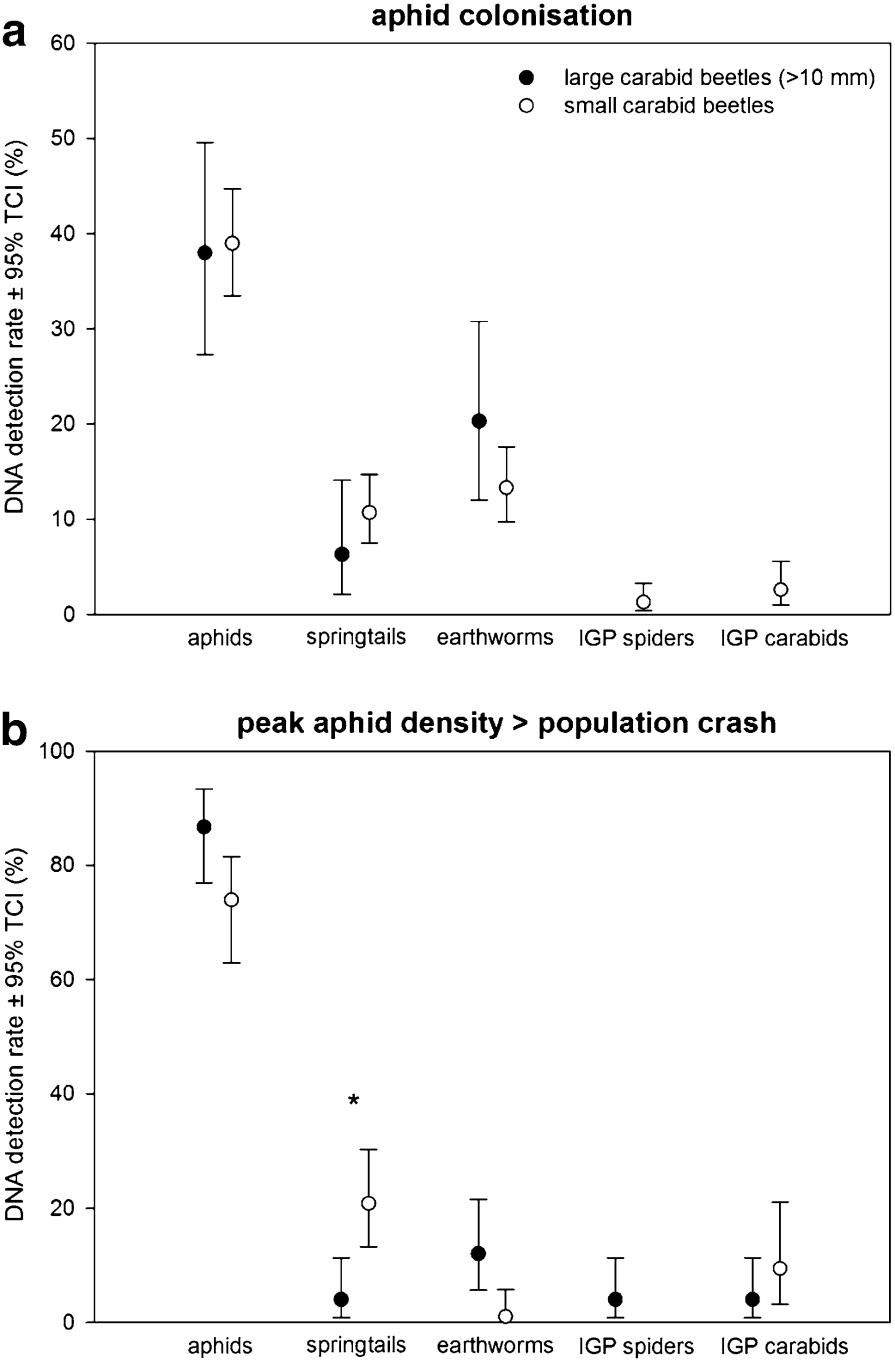
Pooled prey DNA detection rates for aphids, alternative prey groups, and intraguild prey (IGP) in carabid beetles collected in two barley fields in Southern Sweden at **a** aphid colonisation (large, *N* = 78 and small, *N* = 309 beetles) and **b** peak aphid density/population crash (large, *N* = 75 and small, *N* = 96 carabid beetles). *Asterisk* indicates significantly different DNA detection rates between large and small carabid beetles [*P* < 0.05, as tilting confidence intervals (TCI) are not overlapping]. Note that non-detected prey taxa are not shown and that the values for intraguild predation of spiders and carabids are pooled detections of MPII spiders and MPII beetles/thrips, respectively

Prey DNA could be amplified in 62.5 % of the 558 specimens analysed (by size class, in 73.9 % of large and 58.3 % of small carabids) with up to three prey types detected per beetle. Aphids were the most frequently detected prey: at aphid colonisation 38 % of large and 39 % of small carabids tested positive for aphid DNA and 86.7 % versus 72.9 % at peak density (two fields pooled, Fig. 3). As the availability of aphids in the two fields differed dramatically later in the season (see “Materials and methods”), we also assessed DNA detection rates for aphids and other prey types separately for each field for the second sampling date. Approximately 60 % of both large and small carabids tested positive for aphid DNA in field B, whereas as many as 96.4 % of large and 85.7 % of small carabids tested positive for aphid DNA in the highly infested field A (ESM 4). Alternative prey was consumed to a smaller extent with a total DNA detection rate below 21 % in all cases and relative rates remained similar despite pronounced differences in aphid densities at the second sampling date (Fig. 3, ESM 4). The proportion of carabid beetles testing positive for DNA of earthworms at the first sampling date was 20.3 % in large carabids and 13.3 % in smaller ones. Springtail DNA detection rate was 6.3 and 10.7 % in large and small carabids, respectively. At the second sampling date, springtail DNA detection rates were significantly higher in small carabids (20.8 %) compared to larger carabids (4 %) (*P* < 0.05), but there was no such pronounced difference for earthworm prey (Fig. 3). Intraguild predation on spiders (max. 5 % in large carabids, field B, second sampling date) and between carabids (max. 17 % in small carabids field B, second sampling date) was generally low and at the first sampling date only small carabids tested positive for DNA of intraguild prey. DNA of the ladybeetle *C. septempunctata* and thrips was only rarely detected (<2.5 %, ESM 3); whereas DNA of dipterans, lacewings, *Pachygnatha* spp., and *Pterostichus* spp. was not detected at all.

## Discussion

We present new prey-specific and highly sensitive PCR primers to effectively assess predator–prey trophic interactions in cereal crops. With the specific purpose of making the presented molecular detection system available as a ‘ready to use’ approach, these primers have been herein combined into three multiplex PCR assays. This offers a quick and cost-effective screening of large numbers of predator samples: for example, the DNA extracts of the 558 field-collected carabid beetles examined were screened in three days by a single person. The newly developed primers address economically important cereal aphid species (*R.padi*, *Sitobionavenae,* and *Metopolophium dirhodum*), as well as non-aphid extraguild prey such as springtails, earthworms, and dipterans, and intraguild prey including ground-dwelling generalist predators (beetles and spiders) and aphidophagous specialist predators (ladybeetles, lacewings). All of the addressed invertebrate taxa are common to temperate agricultural systems, and while these molecular assays were designed for cereal systems they are by no means restricted to these but could easily be applied to unravel food web interactions in a range of arable crops. We also want to emphasise that the developed multiplex PCR assays could, with little work, be adapted to cover those prey taxa which are of interest to a particular study. For example, such that only a selection of the presented primers, or primers specific to prey taxa not covered, such as parasitoids [e.g. see primers by Traugott et al. (2012) for *Aphidius/Ephedrus/Dendrocerus* spp.] or hoverflies [Diptera: Syrphidae; see primers developed by Gomez-Polo et al. (2014) and Sint et al. (2014)] could be included. For this study, all primers have been extensively tested, however, in any system where novel taxa are present, we strongly recommend evaluating the primers’/assays’ specificity a priori. Likewise, in any case where novel prey-specific primers should be combined with the ones presented here, this evaluation step is necessary to assure reliable results.

We initially evaluated some of the published primers for prey groups, e.g. aphids or dipterans, in silico to check for a possible use in our assays. However, they did not fully meet our requirements. For example, group-specific primers either did not provide full coverage of the entire group and/or did not ensure specificity for all taxa we were interested in (e.g. aphid primers, Harper et al. 2005; Diptera primers, King et al. 2011). Other primers were designed on different genes, e.g. the mitochondrial COII/12S rRNA genes or the nuclear ITS-1 region (e.g. aphid primers, Chen et al. 2000; earthworm primers, Harper et al. 2005; *C. septempunctata* primers, Gagnon et al. 2011) for which we had not established the respective DNA sequence databases which would be needed for rigorous in silico evaluations.

A further output of this study is that a set of DNA sequences (both 18S and COI) for arthropods commonly found in agricultural systems has been generated and made publicly available (ESM 1; GenBank accession numbers: KT204317–KT204433). New sequences include for instance, 18S-sequences for *P. versicolor* and *Erigone atra* and COI sequences for some of the agriculturally important spiders of the family *Pardosa* (i.e. *P. palustris, P. prativaga*), the staphylinid beetle *Atheta gregaria,* and the thrips *L. denticornis*. These DNA sequences can be used to develop further prey-specific primers (e.g. species-specific primers for spiders) to be combined in new customised multiplex PCR assays (see above). Furthermore, sequence database-dependent approaches such as DNA barcoding (Hebert et al. 2003) and NGS-based prey identification techniques (Shokralla et al. 2012, Pompanon et al. 2012) will benefit from the extended number of DNA sequences.

In a first test of applicability, our three multiplex PCR assays proved highly efficient for MGCA of field-collected carabid beetles. The outcomes of this screening are consistent with the role of carabids as natural enemies of aphids in the early stage of pest population development (Chiverton 1987; Lang 2003; Östman et al. 2003). Here aphid DNA detection rates were approx. 40 % in both fields and were clearly exceeding all other prey types we were testing for. Later in the season, aphid DNA was even more frequently detected, i.e. >95 % of the collected large carabids tested positive in the highly infested barley field. Note that the *per capita* predation rate on aphids cannot be precisely quantified using MGCA, but it provides a proxy of the trophic interaction strength (Symondson 2012). All of the collected carabid species tested positive for aphid DNA, except for five species where fewer than six individuals were caught, and there were no significant differences in aphid DNA detection rates between large and small beetles. This suggests that aphids are a frequently used prey in arable carabid communities and that conservation efforts for these beetles are worthy of pursuit (Collins et al. 2002; Ekbom and Wiktelius 1985; Rusch et al. 2013).

Alternative extraguild prey such as earthworms and springtails were consumed to a much smaller extent, indicating little distraction of predators from feeding on aphids in these cereal fields. We refrain from drawing further conclusions, as the availability of alternative prey in the plots has not been estimated here. As for the beetles’ body size, the results are in accordance with studies showing that it is closely related with the size of their preferred prey (Kalinkat et al. 2011; Schneider et al. 2012; Wheater 1988): springtail DNA detection rate was significantly higher in small carabids such as *Bembidion* spp., whereas larger carabids (e.g. *Poecilus* spp. and *Pterostichus* spp.) tested positive for earthworm DNA. This taxon, on average, should constitute a larger prey than springtails. Furthermore, our screening revealed a generally low frequency of intraguild prey DNA detection in the examined carabids, which indicates that antagonistic effects among predators might be playing a minor role in the investigated barley fields.

Summarising, the new molecular assays presented here offer a quick and straightforward approach for assessing previously cryptic trophic interactions between generalist predators and their potential prey, particularly cereal aphids. This will allow adopting food web approaches and thus lead to a better mechanistic understanding of biological control of agricultural pests (Griffin et al. 2013; Rusch et al. 2014; Tixier et al. 2013).

## Author contribution statement

KS, MJ, and MT conceived and designed the study. KS designed the primers, established the molecular assays, and performed the laboratory work. The field collection of carabid beetles in Southern Sweden was accomplished by KS with the support of several field assistants. KS analysed the data, compiled tables and figures, and wrote the paper. MT and MJ revised and improved the manuscript.

## Electronic supplementary material

Supplementary material 1 (DOCX 185 kb)
