# Supplementary material for: Diagnostic PCR assays to unravel food web interactions in cereal crops with focus on biological control of aphids
Source: J Pest Sci (2004). 2015 Aug 11;89:281–93. doi: 10.1007/s10340-015-0685-8 (PMC4757624; doi:10.1007/s10340-015-0685-8)
Supplement: Supplementary file 1 — Supplementary material 1 (DOCX 185 kb) [file 10340_2015_685_MOESM1_ESM.docx]

**Electronic Supplementary Material**

**ESM 1** GenBank accession numbers of representative DNA sequences of target and non-target taxa. Columns show the taxonomic affiliation of the invertebrate taxa, the gene for which sequences are provided and an indication of whether the sequences have been generated for this study (the identity of all taxa was assured by qualified taxonomists) or retrieved from GenBank

| **taxa** | **GenBank accession nos** | **gene** | **present study** |
| --- | --- | --- | --- |
| Carabidae, *Acupalpus* *parvulus* | JN619253 | 18S |  |
|  | KT204374 | COI | **🗸** |
| Carabidae, *Anchomenus dorsale* | KT204317 | 18S | **🗸** |
|  | KT204375 | COI | **🗸** |
| Carabidae, *Agonum muelleri* | KT204318 | 18S | **🗸** |
|  | KT204376 | COI | **🗸** |
| Carabidae, *Amara aenea* | FJ173123 | 18S |  |
| Carabidae, *Amara bifrons* | FN868610 | COI |  |
| Carabidae, *Amara similata* | KT204377 | COI | **🗸** |
| Carabidae, *Asaphidion flavipes* | KT204319 | 18S | **🗸** |
|  | KT204378 | COI | **🗸** |
| Carabidae, *Bembidion lampros* | KT204320 | 18S | **🗸** |
|  | KT204379 | COI | **🗸** |
| Carabidae, *Bembidion quadrimaculatum* | KT204321 | 18S | **🗸** |
|  | KT204380 | COI | **🗸** |
| Carabidae, *Bembidion tetracolum* | AF201402 | 18S |  |
|  | KT204381 | COI | **🗸** |
| Carabidae, *Bembidion guttula* | KT204322 | 18S | **🗸** |
|  | KT204382 | COI | **🗸** |
| Carabidae, *Calathus fuscipes* | FJ173118 | 18S |  |
| Carabidae, *Calathus melanocephalus* | KJ962712 | COI |  |
| Carabidae, *Carabus cancellatus* | JX279744 | 18S |  |
| Carabidae, *Carabus granulatus* | KT204383 | COI | **🗸** |
| Carabidae, *Clivina fossor* | KT204323 | 18S | **🗸** |
|  | KT204384 | COI | **🗸** |
| Carabidae, *Harpalus affinis* | KT204324 | 18S | **🗸** |
|  | KT204385 | COI | **🗸** |
| Carabidae, *Harpalus rufipes* | KT204325 | 18S | **🗸** |
|  | KT204386 | COI | **🗸** |
| Carabidae, *Loricera* *pilicornis* | AF201396 | 18S |  |
|  | KT204387 | COI | **🗸** |
| Carabidae, *Nebria brevicollis* | AF201395 | 18S |  |
|  | KT204388 | COI | **🗸** |
| Carabidae, *Patrobus longicornis* | AF002786 | 18S |  |
| Carabidae, *Poecilus cupreus* | KT204326 | 18S | **🗸** |
|  | KT204389 | COI | **🗸** |
| Carabidae, *Poecilus versicolor* | KT204327 | 18S | **🗸** |
|  | KT204390 | COI | **🗸** |
| Carabidae, *Pterostichus melanarius* | KT204328 | 18S | **🗸** |
|  | KT204391 | COI | **🗸** |
| Carabidae, *Pterostichus niger* | KT204329 | 18S | **🗸** |
|  | KT204392 | COI | **🗸** |
| Carabidae, *Synuchus* *vivalis* | FJ173120 | 18S |  |
|  | KT204393 | COI | **🗸** |
| Carabidae, *Trechus quadristriatus* | KT204330 | 18S | **🗸** |
|  | KT204394 | COI | **🗸** |
| Carabidae, *Trechus secalis* | KT204331 | 18S | **🗸** |
|  | KT204395 | COI | **🗸** |
| Staphylinidae, *Atheta* *gregaria* | KT204332 | 18S | **🗸** |
|  | KT204396 | COI | **🗸** |
| Staphylinidae, *Philonthus fimetarius* | JN619017 | 18S |  |
| Staphylinidae, *Stenus providus* | AY745615 | 18S |  |
| Staphylinidae, *Stenus clavicornis* | KT204397 | COI | **🗸** |
| Staphylinidae, *Tachinus rufipes* | KT204333 | 18S | **🗸** |
|  | KT204398 | COI | **🗸** |
| Staphylinidae, *Tachyporus chrysomelinus* | KT204334 | 18S | **🗸** |
|  | KT204399 | COI | **🗸** |
| Staphylinidae, *Tachyporus formosus* | AJ293031 | COI |  |
| Staphylinidae, *Tachyporus hypnorum* | KT204336 | 18S | **🗸** |
|  | KT204400 | COI | **🗸** |
| Staphylinidae, *Tachyporus obtusus* | KT204335 | 18S | **🗸** |
| Staphylinidae, *Xantholinus* *tricolor* | KT204337 | 18S | **🗸** |
|  | KT204401 | COI | **🗸** |
| Coccinellidae, *Adalia bipunctata* | KT204338 | 18S | **🗸** |
|  | KT204402 | COI | **🗸** |
| Coccinellidae, *Adalia decempunctata* | EF512323 | 18S |  |
|  | JQ757053 | COI |  |
| Coccinellidae, *Anatis* *ocellata* | GU073676 | 18S |  |
|  | GU073920 | COI |  |
| Coccinellidae, *Anisosticta* *novedecimpunctata* | AY748146 | 18S |  |
| Coccinellidae, *Coccinella septempunctata* | KT204339 | 18S | **🗸** |
|  | KT204403 | COI | **🗸** |
| Coccinellidae, *Exochomus quadripustulatus* | GU073721 | 18S |  |
|  | AJ429493 | COI |  |
| Coccinellidae, *Harmonia* *axyridis* | GU073689 | 18S |  |
|  | HQ978630 | COI |  |
| Coccinellidae, *Propylea quatuordecimpunctata* | KT204340 | 18S | **🗸** |
|  | KT204404 | COI | **🗸** |
| Chrysomelidae, *Phyllotreta striolata* | FJ973971 | 18S |  |
| Chrysomelidae, *Phyllotreta undulata* | KT204341 | 18S | **🗸** |
|  | KT204405 | COI | **🗸** |
| Chrysomelidae, *Phyllotreta vittula* | KT204342 | 18S | **🗸** |
|  | KT204406 | COI | **🗸** |
| Chrysomelidae, *Psylliodes brettinghami* | FJ973976 | 18S |  |
| Elateridae, *Agriotes obscurus* | HQ333805 | 18S |  |
|  | HM542030 | COI |  |
| Lycosidae, *Alopecosa cuneata* | KT204343 | 18S | **🗸** |
|  | KT204407 | COI | **🗸** |
| Lycosidae, *Alopecosa trabalis* | KT204408 | COI | **🗸** |
| Lycosidae, *Alopecosa virgata* | JN816762 | 18S |  |
| Lycosidae, *Pardosa agrestis* | KT204344 | 18S | **🗸** |
|  | KT204409 | COI | **🗸** |
| Lycosidae, *Pardosa amenata* | KT204345 | 18S | **🗸** |
|  | KT204410 | COI | **🗸** |
| Lycosidae, *Pardosa palustris* | KT204346 | 18S | **🗸** |
|  | KT204411 | COI | **🗸** |
| Lycosidae, *Pardosa prativaga* | KT204347 | 18S | **🗸** |
|  | KT204412 | COI | **🗸** |
| Lycosidae, *Pardosa nigra* | JQ746513 | COI |  |
| Lycosidae, *Pirata piraticus* | HQ924465 | COI |  |
| Lycosidae, *Pirata procurvus* | JN816771 | 18S |  |
| Lycosidae, *Trochosa ruricola* | KT204348 | 18S | **🗸** |
|  | KT204413 | COI | **🗸** |
| Lycosidae, *Trochosa spinipalpis* | KT204349 | 18S | **🗸** |
|  | KT204414 | COI | **🗸** |
| Lycosidae, *Trochosa terricola* | KT204350 | 18S | **🗸** |
|  | KT204415 | COI | **🗸** |
| Linyphiidae, *Araeoncus humilis* | KT204351 | 18S | **🗸** |
| Linyphiidae, *Bathyphanthes gracilis* | KT204352 | 18S | **🗸** |
|  | KT204416 | COI | **🗸** |
| Linyphiidae, *Diplocephalus christatus* | GU338490 | 18S |  |
|  | HQ924452 | COI |  |
| Linyphiidae, *Erigone atra* | KT204353 | 18S | **🗸** |
|  | KT204417 | COI | **🗸** |
| Linyphiidae, *Erigone dentipalpis* | KT204354 | 18S | **🗸** |
|  | KT204418 | COI | **🗸** |
| Linyphiidae, *Agyneta rurestris* | KT204355 | 18S | **🗸** |
|  | KT204419 | COI | **🗸** |
| Linyphiidae, *Oedothorax apicatus* | KT204356 | 18S | **🗸** |
|  | KT204420 | COI | **🗸** |
| Linyphiidae, *Oedothorax retusus* | KT204357 | 18S | **🗸** |
|  | KT204421 | COI | **🗸** |
| Linyphiidae, *Oedothorax fuscus* | KT204358 | 18S | **🗸** |
| Linyphiidae, *Porrhomma microphthalmum* | KT204359 | 18S | **🗸** |
|  | KT204422 | COI | **🗸** |
| Linyphiidae, *Tenuiphanthes* sp. | GU338514 | 18S |  |
| Linyphiidae, *Tenuiphanthes tenuis* | KT204423 | COI | **🗸** |
| Linyphiidae, *Walckenaeria clavicornis* | GU338483 | 18S |  |
| Linyphiidae, *Walckenaeria palustris* | GU683830 | COI |  |
| Tetragnathidae, *Pachygnatha clercki* | KT204360 | 18S | **🗸** |
|  | KT204424 | COI | **🗸** |
| Tetragnathidae, *Pachygnatha degeeri* | KT204361 | 18S | **🗸** |
|  | FJ899819 | COI |  |
| Tetragnathidae, *Tetragnatha extensa* | GU684028 | COI |  |
| Tetragnathidae, *Tetragnatha maxillosa* | AY425723 | 18S |  |
| Theridiidae, *Theridion impressum* | KT204425 | COI | **🗸** |
| Thomisidae, *Xysticus* *obscurus* | KF369067 | COI |  |
| Thomisidae, *Xysticus* *sicus* | JN816831 | 18S |  |
| Aphididae*, Metopolophium dirhodum* | KT204362 | 18S | **🗸** |
|  | KT204426 | COI | **🗸** |
| Aphididae*, Rhopalosiphum padi* | KT204363 | 18S | **🗸** |
|  | KT204427 | COI | **🗸** |
| Aphididae*, Sitobion avenae* | KT204364 | 18S | **🗸** |
|  | KT204428 | COI | **🗸** |
| Aphididae*, Chaitophorus capreae* | HM988752 | 18S |  |
| Lumbricidae*, Allolobophora chlorotica* | HM417954 | COI |  |
| Lumbricidae*, Aporrectodea* *caliginosa* | JQ908896 | COI |  |
| Lumbricidae*, Aporrectodea* *trapezoides* | HQ621897 | 18S |  |
| Lumbricidae*, Dendrobaena clujensis* | AJ272527 | 18S |  |
| Lumbricidae*, Dendrobaena octaedra* | JQ909051 | COI |  |
| Lumbricidae*, Dendrodrilus* *rubidus* | GU901868 | 18S |  |
|  | JQ909082 | COI |  |
| Lumbricidae*, Eisenia andrei* | AY874511 | COI |  |
| Lumbricidae*, Eisenia fetida* | AB558505 | 18S |  |
| Lumbricidae*, Lumbricus* *terrestris* | HQ691211 | 18S |  |
|  | JQ909131 | COI |  |
| Lumbricidae*, Octolasium lacteum* | AJ272312 | 18S |  |
| Collembola, *Cryptopygus caecus* | HQ592688 | COI |  |
| Collembola, *Entomobrya dorsosignata* | AY596360 | 18S |  |
| Collembola, *Entomobrya nivalis* | HG422599 | COI |  |
| Collembola, *Folsomia candida* | AY555515 | 18S |  |
| Collembola, *Folsomia* sp. | HG422608 | COI |  |
| Collembola, *Isotomiella minor* | HG422636 | COI |  |
| Collembola, *Isotoma riparia* | HG422621 | COI |  |
| Collembola, *Isotoma viridis* | AY596361 | 18S |  |
| Collembola, *Isotomurus palustris* | DQ016560 | 18S |  |
| Collembola, *Onychiurus yodai* | AY037171 | 18S |  |
| Collembola, *Parisotoma notabilis* | JQ935202 | COI |  |
| Collembola, *Sminthurus viridis* | AY859604 | 18S |  |
| Collembola, *Sminthurinus elegans* | JQ909238 | COI |  |
| Syrphidae, *Episyrphus balteatus* | KT204365 | 18S | **🗸** |
|  | KT204429 | COI | **🗸** |
| Syrphidae, *Eristalis arbustorum* | KT204366 | 18S | **🗸** |
|  | KT204430 | COI | **🗸** |
| Syrphidae, *Eristalis tenax* | JN991985 | COI |  |
| Syrphidae, *Helophilus* *hybridus* | KT204367 | 18S | **🗸** |
| Syrphidae, *Scaeva pyrastri* | EU431553 | 18S |  |
|  | JN992029 | COI |  |
| Syrphidae, *Sericomyia silentis* | KT204368 | 18S | **🗸** |
| Syrphidae, *Sericomyia chrysotoxoides* | JF442710 | COI |  |
| Syrphidae, *Sphaerophoria* *scripta* | EU241860 | 18S |  |
| Syrphidae, *Syrphus vitripennis* | HQ845768 | 18S |  |
| Anthomyiidae, Anthomyiidae sp. | HQ979118 | COI |  |
| Calliphoridae, *Calliphora nigribarbis* | AB466039 | 18S |  |
| Calliphoridae, Calliphoridae sp. | KC135914 | COI |  |
| Chironomidae, Chironomidae sp. | HQ979200 | COI |  |
| Dolichopodidae, Dolichopodidae sp. | GU013594 | COI |  |
| Drosophilidae, *Drosophila melanogaster* | HM102299 | COI |  |
| Empididae, Empididae sp. | HQ939431 | COI |  |
| Muscidae, Muscidae sp. | JF870659 | COI |  |
| Sciaridae, Sciaridae sp. | HQ979047 | COI |  |
| Tipulidae, *Dolichopeza subalbipes* | AY521834 | 18S |  |
| Tipulidae, Tipulidae sp. | KC136018 | COI |  |
| Chrysopidae, *Chrysoperla carnea* | KT204369 | 18S | **🗸** |
|  | KT204431 | COI | **🗸** |
| Chrysopidae, *Chrysoperla plorabunda* | L10183 | 18S |  |
| Thysanoptera, *Frankliniella intonsa* | KT204370 | 18S | **🗸** |
|  | HM246175 | COI |  |
| Thysanoptera, *Frankliniella occidentalis* | KC512959 | 18S |  |
|  | JX235929 | COI |  |
| Thysanoptera, *Limothrips denticornis* | KT204371 | 18S | **🗸** |
| Thysanoptera, *Aeolothrips fasciatus* | KT204372 | 18S | **🗸** |
|  | KT204432 | COI | **🗸** |
| Thysanoptera, *Anaphothrips incertus* | KC512926 | 18S |  |
| Thysanoptera, *Anaphothrips obscurus* | HM246168 | COI |  |
| Thysanoptera, *Haplothrips graminis* | KC512965 | 18S |  |
|  | KC513158 | COI |  |
| Thysanoptera, *Haplothrips aculeatus* | HQ605967 | COI |  |
| Braconidae, *Aphidius rhopalosiphi* | KT204373 | 18S | **🗸** |
|  | KT204433 | COI | **🗸** |
| Braconidae, *Ephedrus persicae* | AJ009329 | 18S |  |

**ESM 2** Further details on the molecular approach, prey-specific primers, and customised multiplex PCR assays

*Standard singleplex PCR protocol for amplification of 18S and COI DNA*

These PCRs were performed in 10 µl reactions containing 1.5 µl of DNA extract, 0.25 U One*Taq^®^* DNA polymerase (NEB, Ipswich, USA), 1× reaction buffer (NEB) and additional MgCl_2_ to a final concentration of 3 mM, 0.2 mM dNTPs (Genecraft, Köln, Germany), 5 µg bovine serum albumin (BSA), 1 µM of each primer, and PCR-grade water to adjust the volume. Amplifications were carried out under the following thermocycling conditions: initial denaturation of 2 min at 94°C, 35 cycles of 20 s at 94°C, 30 s at 50°C, and 1 min at 68°C followed by a final elongation of 3 min at 68°C. Note that this protocol was used for amplification of the 18S and COI gene for subsequent DNA sequencing, for DNA template generation (sensitivity tests) and also to check extraction negative controls as well as ‘screening-negatives’ (two carabid DNA extracts) with the universal primers.

*Evaluation of newly developed primers in singleplex PCR*

The specificity and sensitivity/diagnostic efficacy of all primer pairs were evaluated in singleplex PCRs based on the optimized multiplex PCR protocols (Qiagen, see Results). Those employed in one of the three multiplex PCR assays were tested using the respective conditions of the multiplex PCR protocol, with the exception of a modification in primer concentration of 0.5 µM and annealing temperature of 62°C. The primers not included in multiplex PCR assays were tested as follows: the ladybeetle primer pair and the second primer versions for aphids, springtails, and dipterans were tested with the *MPI* protocol, the second version for *Pachygnatha* spp. with the *MPII spiders,* and the primer pair for *Trechus* spp. and second versions for *Harpalus* spp. and *C. septempunctata* with the *MPII beetles/thrips* protocol.

*Beetle/thrips-primers*

A second version of the beetles/thrips-forward primer which perfectly matches ladybeetles (S405.1) was designed and we suggest using 1:1 mixes of the two primers A405 and A405.1 if an inclusion of ladybeetles is desired. Note that a 10–15 bp shorter fragment was amplified in PCR with DNA of rove-, lady-, leafbeetles, and thrips compared to DNA of carabid beetles which is due to gaps in the respective region of the 18S gene. DNA of the carabid beetle *Nebria* sp. could not be amplified because of mismatches at the 3’ end of the forward primer. This is also true for the two tested elaterids, *Agriotes obscurus* and *Hemicrepidius niger.* Note that DNA of Cantharidae, Silphidae, and the ladybeetle *Exochomus* sp. could not always be amplified – most likely due to similar deficiencies of these group-specific primers.

*Genus-specific primers for Carabidae*

The genus-specific primers for *Poecilus* spp. were designed based on the COI gene and are thus most likely *P. cupreus/versicolor*-specific; DNA of *Poecilus sericeus* (the only other species tested in PCR) could not be amplified. On the contrary, the 18S-based primers for *Pterostichus* spp. and *Bembidion* spp. might also work for congeners as primers perfectly fit *P. illigeri* when tested *in silico* and DNA of *B. guttula* and *B. properans* was successfully amplified in PCR. The primers for *Harpalus* spp. (version 1) should also work for *H. aenaeus* (tested *in silico* only). The second primer pair for *Harpalus* spp., however, might only be used for the species *H. rufipes* – DNA of the closely-related *H. affinis* was not always amplified in PCR due to mismatches of the reverse primer (A476) for *Harpalus* spp. (version 2).

It was not always possible to ‘exclude’ closely-related taxa when designing these genus-specific primers: the COI-based forward (S475) and reverse primer (A486) for *Poecilus* spp. also fit on *Pterostichus* spp. and *Bembidion* spp., respectively. No cross-reactions could be observed, though, when using these primers in singleplex PCR as the respective other primer is specific in each case. Moreover, in the multiplex PCR assay *MPII beetles/thrips* both *Pterostichus* spp. and *Bembidion* spp. are targeted on the 18S gene making undesired amplifications impossible. Likewise, the use of the reverse primer (A467.1) for *Pterostichus* spp., which perfectly fits on *Harpalus* spp. and *Poecilus* spp., doesn’t constitute a problem in *MPII beetles/thrips*, as both of the latter were based on the COI gene.

We suggest mixing the two versions of the forward primer for *Pterostichus* spp. to achieve amplification of both species *P. melanarius* (S467) and *P. niger* (S467.1). The two closely-related genera *Bembidion* and *Trechus* (both subfamily Trechinae) share the same forward primer (S468); note also that the combination for *Trechus* spp. amplifies a shorter amplicon length for *T. quadristriatus* (142 bp) compared to *T. secalis* (152 bp) due to a gap in the respective region on 18S in the former (Table 2).

*Thrips-primers*

Note that the so-called group-specific primer pair for thrips most probably amplifies DNA of *Frankliniella* spp. and *Limothrips denticornis* only (both species are commonly found in spring-sown cereals). Other genera within this highly diverse group (e.g. *Aeolothrips*, *Haplothrips*, *Anaphothrips*) could not be included.

*Spider- and lycosid-primers*

DNA of tetragnathid spiders produced a slightly longer (~10 bp) fragment compared to other spider families in PCR with the group-specific spiders-primers. DNA of the spider family Theridiidae did not always amplify – however, a longer fragment of ~430 bp was detected when using the *MPI* assay (not observed in singleplex PCR). Note that the so-called family-specific primers for lycosids (primarily designed for *Pardosa/Trochosa/Alopecosa*) might also amplify DNA of *Rabidosa* and *Lycosa* as seen *in silico*; DNA of *Pirata* sp., for which DNA extracts were available, did not produce amplicons in PCR, though.

*Springtail-primers*

When testing the specificity of the group-specific primers for springtails it was not known which species were used in PCR. We have, however, included all of the important taxa within Arthropleona (e.g. *Folsomia* sp., *Entomobrya* sp., *Onychiurus* sp., *Isotoma* sp., *Isotomurus* sp., *Cryptopygus* sp., *Parisotoma* sp., and others) and *Sminthurus* sp. (Symphypleona) in the 18S sequence alignment for *in silico* evaluation.

*Dipteran-primers*

Note that the aphidophagous hoverflies (Diptera: Syrphidae) are also covered by the group-specific primers for dipterans. If disentanglement is desired, family-specific primers as developed by Gomez-Polo et al. (2014) and Sint et al. (2014) might be used subsequently. Our *in silico* evaluations showed that all of the three primers for dipterans (S413, S414, A416) have several deficiencies regarding the family of Chironomidae. Only the dipterans2-primers did sometimes amplify Chironomidae DNA in PCR.

**ESM 3** Carabid beetles collected in two barley fields in Southern Sweden in spring 2012 and subjected to molecular gut content analysis. Columns show the species and body size allocation of the carabids (L, large, i.e., >10 mm; S, small), the sampling date (May: aphid colonisation and/or June: peak aphid density/population crash) and field (A and/or B) where a specific carabid species was found and number of specimens collected (sampling dates and fields pooled). The prey DNA detection per species is provided as number of individuals testing positive for each prey taxon targeted in the three multiplex PCR assays. IGP refers to intraguild prey; note that the values for intraguild predation of spiders and carabids are pooled detections of MPII spiders (2 families, 1 genus) and MPII beetles/thrips (4 carabid genera), respectively

| **species** | **size** | **sampling dates** | **fields** | **number of specimens collected** | **aphid DNA detection** | **springtail DNA detection** | **earthworm DNA detection** | **thrips DNA detection** | **spider DNA detection (IGP)** | **carabid DNA detection (IGP)** | **Coccinella septempunctata DNA detection (IGP)** |
| --- | --- | --- | --- | --- | --- | --- | --- | --- | --- | --- | --- |
| *Acupalpus dorsalis* | S | May | B | 2 | 2 |  |  |  |  |  |  |
| *Acupalpus exiguus* | S | May | B | 1 |  |  |  |  |  |  |  |
| *Anchomenus dorsale* | S | May + June | A + B | 29 | 12 | 1 | 3 |  |  | 2 |  |
| *Agonum muelleri* | S | May + June | A + B | 11 | 6 | 1 | 2 |  |  | 2 |  |
| *Amara aenea* | S | May | B | 6 | 2 |  |  |  |  | 2 |  |
| *Amara apricaria* | S | June | B | 1 | 1 |  |  |  |  |  |  |
| *Amara curta* | S | June | B | 1 | 1 |  |  |  |  |  |  |
| *Amara plebeja* | S | May+June | A+B | 13 | 9 | 3 |  |  | 1 | 2 | 1 |
| *Amara tibialis* | S | May+June | B | 2 | 1 |  |  |  |  | 1 |  |
| *Asaphidion flavipes* | S | May | B | 5 | 1 | 1 | 1 |  |  |  |  |
| *Bembidion lampros* | S | May+June | A+B | 171 | 91 | 33 | 9 |  | 1 | 1 | 1 |
| *Bembidion obtusum* | S | May | B | 1 |  |  |  |  |  |  |  |
| *Bembidion tetracolum* | S | May+June | A+B | 129 | 41 | 8 | 23 |  | 1 |  |  |
| *Calathus melanocephalus* | S | May+June | A+B | 6 | 5 | 2 |  |  |  |  |  |
| *Clivina fossor* | S | May+June | A+B | 23 | 18 | 4 | 2 |  | 1 | 2 |  |
| *Harpalus affinis* | L | May+June | A+B | 12 | 5 | 1 | 2 |  | 1 | 1 |  |
| *Harpalus distinguendus* | L | May+June | B | 3 | 2 |  | 1 |  |  | 1 |  |
| *Harpalus rufipes* | L | May+June | A+B | 13 | 5 | 2 |  |  |  | 1 |  |
| *Loricera pilicornis* | S | May+June | B | 5 |  |  | 2 |  |  |  |  |
| *Nebria brevicollis* | L | May | B | 8 | 1 |  | 3 | 1 |  |  |  |
| *Poecilus cupreus* | L | May | A+B | 26 | 7 | 2 | 9 |  |  |  | 1 |
| *Poecilus lepidus* | L | June | B | 1 |  |  |  |  |  |  |  |
| *Poecilus versicolor* | L | May+June | A+B | 30 | 21 |  | 3 |  |  |  |  |
| *Pterostichus diligens* | L | May | B | 2 | 1 |  |  |  |  |  |  |
| *Pterostichus melanarius* | L | May+June | A+B | 58 | 53 | 3 | 6 |  | 2 |  |  |
| *Pterostichus niger* | L | June | B | 1 |  |  | 1 |  |  |  |  |

**ESM 4** Pooled prey DNA detection rates for aphids, alternative prey groups, and intraguild prey (IGP) in carabid beetles collected in Southern Sweden in late June 2012 in two barley fields: **a,** field A, where aphid population had reached its peak density of approx. 30 aphids per tiller (large, N=55 and small, N=49 carabid beetles) and **b,** field B, where aphid population had already crashed leaving less than one aphid per tiller (large, N=20 and small, N=47 carabid beetles). Asterisk indicates significantly different DNA detection rates in large and small carabid beetles (*P*<0.05, as tilting confidence intervals [TCI] are not overlapping). Note that non-detected prey taxa are not shown and that the values for intraguild predation of spiders and carabids are pooled detections of MPII spiders and MPII beetles/thrips, respectively

**
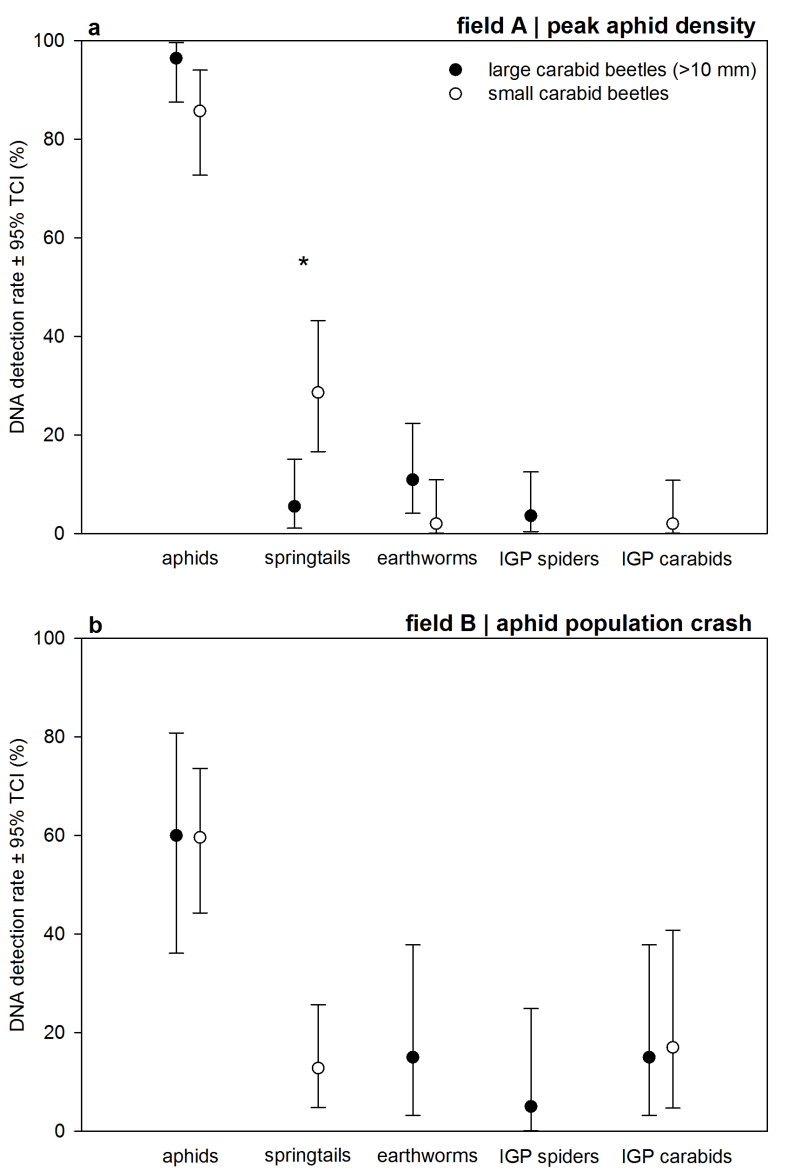
**
